# E Pluribus, Unum: Emergent Redox Harmony from the Chaos of Blood Cells

**DOI:** 10.3390/antiox13091151

**Published:** 2024-09-23

**Authors:** Alkmini T. Anastasiadi, Angelo D’Alessandro

**Affiliations:** 1 Department of Biochemistry, School of Medicine, University of Patras, 26504 Patras, Greece; 2 Department of Biochemistry and Molecular Genetics, Anschutz Medical Campus, University of Colorado Denver, Aurora, CO 80045, USA

Blood cells play a fundamental role in maintaining systemic homeostasis, by responding dynamically to various physiological and environmental stimuli. Nevertheless, they are continuously exposed to stressors such as hypoxia, inflammation, and oxidative agents, which influence not only their function and survival but also their interactions with other cell types and their contributions to systemic processes. The articles amassed in this Special Issue explore some of the myriad ways in which oxidative stress influences blood cell functionality, starting a journey through the labyrinth of redox balance, from the microscopic to the systemic, and back again.

Redox equilibrium is essential from the dawn of life of blood cells until their final moments. The unobstructed progress of hematopoiesis—from stem cell precursors to mature erythrocytes—relies, among other things, on the redox status of the organism [1,2]. When this equilibrium is disrupted, and the scales tilt toward the accumulation of reactive oxygen species (ROS), the ability of hematopoietic stem cells to thrive, proliferate, and differentiate falters beneath the weight of oxidative stress [3]. Mbiandjeu et al. [Contribution 1] shine a light on this delicate balance by presenting evidence that the nuclear factor erythroid-derived 2 (Nrf2) regulates the antioxidant response during erythropoiesis, while dysfunction in this pathway leads to severe oxidative lesions, premature death of erythroblasts and overall ineffective erythropoiesis. The authors show that this perturbation is attenuated upon antioxidant supplementation, a finding analogous to that of Bo et al. [Contribution 2], who revealed that ascorbic acid seems to protect bone marrow cells from excessive oxidative stress generated by X-ray irradiation. The ability of this simple molecule to restore white blood cell counts upon supplementation in knockout rodent models underscores the profound interdependence between antioxidant systems and hematopoiesis. This relationship even extends to iron metabolism—a key player in hemoglobin synthesis. More specifically, ascorbic acid indirectly regulates erythropoiesis, by facilitating the uptake of iron from enterocytes [Contribution 3]. The acquired iron is then exported, binds to plasma transferrin, and is supplied to erythroid precursors to proceed with their differentiation.

Oxidative stress is a sword of Damocles for circulating blood cells, whose lifespan in circulation is regulated by the balance between the production of ROS and the body’s ability to neutralize them [4], a process that ultimately leads to cellular damage and dysfunction. A case in point is presented in the study by Chakraborty et al. [Contribution 4], who examined RBCs in twins with discordant birth weights. It was shown that the impaired nitric oxide synthesis aids in the generation of reactive species, which oxidize the RBC membrane and induce morphological alterations in the lower-weight sibling cohort; thus, they potentially exert a significant impact on the physiological status of the vascular system. Autism spectrum disorder (ASD) is another condition in which high systemic oxidative stress is mirrored in the RBC redox status and membrane properties. Given the close association between RBC physiology and function and the high oxygen needs of nerve cells, the deterioration of RBCs could be linked to ASD clinical manifestations [Contribution 5]. Both findings highlight that oxidative stress not only affects immediate cellular function but can also cast long shadows on long-term health outcomes. Of course, oxidative dysregulation might be a result of specific mutations, as in the case of Lesch–Nyhan syndrome, where the deficiency in hypoxanthine–guanine phosphoribosyltransferase fuels the generation of urate, which is accompanied by excessive production of hydrogen peroxide. As thoroughly discussed in this Special Issue [Contribution 6], the RBCs of such subjects are characterized by altered metabolism, elevated oxidative stress markers, and significantly compromised integrity. Notably, treatment with allopurinol only partially alleviates some of the observed imbalances, without fully restoring normal RBC function.

In the face of such bleak prospects, the antioxidant defenses of blood cells are invaluable for the maintenance of redox balance. The cellular network of antioxidant systems includes enzymatic antioxidants such as superoxide dismutase or peroxiredoxin and non-enzymatic antioxidants like glutathione [5]. However, this defense system can be compromised in pathophysiological conditions. For instance, in the context of obesity, when the organism is in a state of constant oxidative stress, RBCs are expected to address increased oxidative insults. As revealed in the study by Szlachta et al. [Contribution 7], oxidative markers are reduced upon weight loss, while antioxidant enzyme activities are differentially affected based on the glycemic status of each participant, highlighting the complex connection between metabolic and antioxidant networks within RBCs, as well as the adaptability of the latter depending on their environment. Based on the observation that redox equilibrium is disrupted in disease states, the promise of redemption lies in therapeutic strategies that can restore redox balance and ultimately improve cellular function [6]. For instance, the high-dose administration of the antioxidant L-glutamine to patients with sickle cell disease alleviates several hallmarks of the disease, including inflammation and coagulation, probably through its redox-restorative potential [Contribution 8]. A similar effect is highlighted with ergothioneine, a dietary molecule that can inactivate several ROS. The comprehensive analysis by Thomas et al. [Contribution 9] pointed to the potential benefits of ergothioneine supplementation for patients with hematological conditions characterized by redox imbalance, due to the ability of ergothioneine to enter erythroid progenitors and exert its antioxidant properties. Moreover, ergothioneine levels could be also associated with the superior storability of RBCs or their post-transfusion outcomes. A similar study [Contribution 10], which focuses on another formed element of blood, namely platelets, further highlights the significance of antioxidant therapies. Cysteine oxoforms are involved in oxidative stress-related thrombotic phenomena via their interaction with platelet receptors and thiol isomerases. Therefore, antioxidant strategies that target oxidative cysteine modification have the potential to prevent thrombosis, as is the case of nucleophile ligands, which can target oxidized cysteines.

In the tale of blood cells, the script of redox balance is all but a linear one. From their birth in the marrow to their final moments in circulation, the crosstalk between 25 trillion blood cells—coming together as a sort of circulating organ [7]—and the remaining 5 trillion cells in the human body, is based upon a stride between the opposing forces of oxidative stress and antioxidant defense. The studies presented in this Special Issue further our understanding of the relationship between oxidative stress and blood cell functionality, offering new insights into the systemic importance of maintaining redox balance and pointing to new frontiers in both research and therapy.
